# Associated Risk Factors of STIs and Multiple Sexual Relationships among Youths in Malawi

**DOI:** 10.1371/journal.pone.0134286

**Published:** 2015-08-06

**Authors:** Wilson Chialepeh N, Sathiyasusuman A

**Affiliations:** Department of Statistics and Population Studies, University of the Western Cape, Cape Town, South Africa; Örebro University, SWEDEN

## Abstract

**Background:**

Having unprotected sex with multiple sexual partners (MSP) is the greatest risk factor for human immunodeficiency virus (HIV) and other sexually transmitted infections (STIs) among youths. Young people with MSPs are less likely to use a condom and the greater the risk for STIs. This study examines the associated risk factors of STIs and multiple sexual partnerships among youths aged 15–24 years.

**Data and Methods:**

The Malawi Demographic Health Survey 2010 data was used. Out of a sample of 2,987 males and 9,559 females aged 15–24 years, 2,026 males and 6,470 females were considered in the study. Chi square test and logistic regression techniques were performed. Analysis was performed using Statistical Package for Social Sciences (SPSS) version 22.

**Findings:**

The results indicate that 1,399 (69.0%) males and 2,290 (35.4%) females reported multiple sexual partnerships (MSP). Within the rural area, females (n = 1779) were more likely to report MSP than males (n = 1082) and within the urban areas, a higher proportion of females (n = 511) still reported MSP, with males (n = 316). About 78% rural females aged 20–24 years, and about 79% rural males aged 15–19 years reported MSP. The likelihood of MSP was higher among females in the poorest households (OR = 1.31), being married (OR = 5.71) and Catholic males (OR = 1.63), who were married (OR = 1.59). Catholic males (OR = 1.82) in the rural areas, who were married (OR = 1.80) and rural females in the northern region (OR = 1.26) were more likely to have MSP. The odds ratios were higher among urban females in the poorest (OR = 3.45) households who were married (OR = 4.22).

**Conclusions:**

Having more than one sexual partner increases the risk of STIs and sexuality education programs should be introduced that emphasize the danger that surrounds MSP.

## Introduction

Sub-Saharan Africa faces by far the highest rate of HIV and other Sexually Transmitted Infections (STIs). Malawi, like any other country in the sub-Saharan region of Africa, has been affected by the epidemic of HIV/AIDS and other STIs, a result of youth’s irresponsible sexual practices [1]. A number of contributing factors have been suggested in an attempt to explain reasons for the high prevalence of STIs, especially HIV/AIDS, in the sub-Saharan regions of Africa. This includes factors such as poverty [2], lack of male circumcision [3], migration [4], untreated sexually transmitted infections [5] and multiple sexual relationships. Due to the severity and the impact of these infections, most research studies have focused on understanding sexual behaviours that increases the risk of STIs transmission in sub-Saharan Africa. Others have argued that, the high prevalence of STIs, especially HIV/AIDS, observed in these regions, are driven by high levels of multiple sexual partnerships [6]. Multiple sexual partnerships (MSPs) are widely believed to be one of the sexual risk behaviours that put young people at risk for HIV transmission and other STIs [7]. However, the impact of multiple sexual partnerships on the incidence of HIV in sub-Saharan African settings has not been tested appropriately.

In Malawi, 16% of young males and only 2% females aged 15–19 years reported having had two or more sexual partners in the last 12 months preceding the survey [8]. It has been evident that young males tend to have more sexual partners than their female counterparts, irrespective of their marital status [9]. Among 15–49 years adults in Malawi, 11% are infected with STIs especially HIV/AIDS [10] and most of these infections (90%) are transmitted through heterosexual contact [11]. These infections affect Malawians disproportionately with the prevalence much higher among sexually active females than their male counterparts [12]. The devastating socio-economic impact of these infections and its increasing spread has stimulated a shift of research from a biomedical to a societal context of sexual behaviour. The sexual activities of youths are characterized by early onset of sexuality, multiple sexual relationships and a low incidence of condom use [13]. These practices therefore facilitate the spread of STIs, especially HIV. Thus, having multiple sexual partners in a dense sexual network increases the risk of STIs by allowing the virus to spread rapidly. In relationships where non-overlapping sequential partners exist, the delay between ending one relationship and starting another one reduces the probability of STI transmission [14].

Certain risk factors such as education, substance use, alcohol consumption and non-use of contraceptives have been identified as being associated with multiple sexual partnerships (MSP) [15]. There is a general conception that alcohol consumption increases the risk of MSP. A study of sexually active youths in Cameroon found that, alcohol use increases the prevalence of MSP [16]. Another study in Nigeria found that, women who drank alcohol were more likely to have multiple sexual partners [17]. Similarly, a systematic review and meta-analysis of African studies found that alcohol consumers were more likely to be HIV positive [18], thus indicating that they were more likely to engage in multiple sexual relationships, and less likely to use condoms consistently. Other conceptions such as educational levels and place of residence have also been associated with MSP. It is believed that, youths with less education, who live in urban areas and are exposed to media at least once a week, are more likely to have more sexual partners than those who are more educated, live in rural areas and are regularly exposed to the media [19]. Education has therefore been regarded as a protective factor against risky sexual behaviours by reducing the rate of STIs transmission. While other studies in sub-Saharan Africa have reported that formal education and exposure to media reduces the likelihood of engaging in MSP among males and females [20, 21], others have found no association between education and extramarital partnerships [22].

Multiple sexual partnerships are characterized by early sexual activities, greater number of lifetime partners, more frequent coitus and unprotected coitus. In addition, those with more than one sexual partner uses condom less frequently than those with only one partner [23, 24] and this facilitates the spread of Sexually Transmitted Infections. Other studies have regarded age at first intercourse as an outcome variable, while others have used it to explain multiple sexual behaviours within different populations [25]. Young males and females enter into multiple sexual relationships in order to have more latitude in choosing who to marry as they become mature. Irrespective of their intention, females need to be more cautious than males when it comes to having multiple sexual partners simultaneously, as it would instead lower their chances of getting married. An individual’s risk of contracting HIV/STIs therefore, depends on his or her own sexual behaviour [26]. Overlapping sexual networks speed up the rate of HIV transmission among sexually active youths and boost the scale of HIV epidemic, especially among those in sub-Saharan Africa [27]. Nevertheless, empirical evidence supporting the importance of concurrency among youths remains weak [28]. Recent studies have found a very strong relationship between people having had more than one sexual partner and living with HIV but found no association between concurrence in men and HIV incidence in women or between concurrency and HIV prevalence among men [29]. Individuals with multiple sexual partners are more likely to be HIV positive, and the risk of HIV and other STIs is significantly higher among those with multiple sex partners compared with those who had one sex partner. Moreover, having a history of STIs, being in short relationships and suspecting your current partner of infidelity, was a contributing factor towards multiple sexual relationships [30]. The occurrence of numerous consecutive relationships in rapid succession suggests considerable transmission potential.

In Lilongwe, the capital city of Malawi, most partnerships are longer and monogamous, with concurrent partnerships being infrequent with long periods of overlap [31]. Consecutive partnerships have short intervening gaps that could facilitate the spread of sexually transmitted infections. Although most health related studies report one partner throughout their survey period among youths, the number of recent partners is not the only determinant of youth’s risky sexual behaviour and STI acquisition. The acquisition of sexually transmitted infections does not depend on one’s own behaviour, but also on one’s partner attitudes [32]. A relatively low risk sexual behaviour in high STI odds may result in a person encountering an STI infected partner. Other contextual factors such as the odds of male circumcision, men having sex with men (MSM) and other STIs odds demonstrate the role of concurrent sexual partnerships in amplifying the spread of HIV/AIDS and other STIs within dense sexual networks [33]. Empirical research has however identified the association between concurrent sexual partnerships and an increased risk of sexually transmitted infections including syphilis and gonorrhoea [34].

Certain cultural practices have emerged as an important factor when studying sexual behaviours in most societies. In the African context, cultural norms permit polygamy and condone male’s promiscuity on the grounds that, male sexual drives cannot be controlled [35]. There is a growing conception that certain cultural norms and social institutions promote and even institutionalize MSPs to be socially accepted as forms of sexual conduct. In African patriarchal society for instance, social institutions encourage the husband’s decision making power, while forcing women to subordinate their interest to their husbands. Thus, sociocultural factors that facilitate the risk for HIV among couples condone male promiscuity in most patriarchal societies. Although living in an urban area has been associated with a higher prevalence of MSP and an increased risk of HIV and other STIs, most studies did not report any significant differences in the sexual behaviour of urban and rural residents [36]. Other studies in Malawi have addressed issues of sexuality, multiple sexual partnerships and STIs among youths, but there is limited evidence of the associated risk factor of STIs and multiple sexual partnerships among youths. This current study therefore tests the hypothesis that multiple sexual relationships are an associated risk factor for STIs among young people, using socio-economic and demographic characteristics.

## Data

The relevant data was extracted from the Malawi Demographic and Health Survey findings (MDHS 2010). The data was downloaded from www.measuredhs.com after receiving permission from ICF Macro International. This data was extracted from a cross-sectional representative survey that was conducted in Malawi from June to November 2010, on different topics using a multistage cluster sample of 27, 345 households [37].

Each district was subdivided into enumeration areas (EAs) referred to as clusters. The sample for the survey was conducted at the district and EA levels, using a stratified, two-stage cluster design, and the primary sampling unit were the enumeration areas. In each sampled enumeration area, the households were the secondary sampling units. A total of 849 EAs and 27,345 households were sampled for the survey, and only men aged 15–54 years and women aged 15–49 years were eligible for the survey.

A standardized structure questionnaire was designed to include HIV modules and was administered to eligible members of the sampled households. These questionnaires were used during the previous surveys (2000 and 2004). The Malawi Health and Sciences Research Committee, the Institute Review Board of ICF Macro, and the Centre for Disease Control and Prevention (CDC) in Atlanta, USA granted ethical clearance for the 2010 survey team [38].

A subsample of a third of the household was selected to conduct HIV testing, thus giving a total of 14, 407 men and women aged 15–54 and 15–49 years respectively. Besides the HIV testing, sexually active individuals were asked to state the total number of life time sexual partners they have had during the last 12 months. Moreover, socio-demographic key indicators such as age, sex, marital status, place of residence, region, religion, education level, and ethnicity were also considered in the questionnaires.

Although the number of explanatory variables for both males and females were not the same, the study focuses on those with more than one sexual partner. For the current study, multiple sexual partnerships were used to describe respondents with more than one sexual partner, and this was derived from total lifetime number of sexual partners.

## Methods

The data was extracted and the relevant sample was weighted according to the design of the 2010 Malawi Demographic and Health Survey in order to obtain a representative sample for the study. Out of a sample of 2987 males and 9559 females aged 15–24 years, 2026 males and 6470 females were considered in the study. The study uses total lifetime number of sexual partners as the dependent variable from the survey data. Having sex with more than one sexual partner was regarded as risky, and was described as ‘multiple sexual partnership’. The dependent variable was coded 1 if respondents have more than one partner and 0 otherwise. Bivariate analysis was used to test for the association between the dependent and categorical variables by using chi square. A binary logistic regression technique was performed to examine the association of socio-economic and demographic characteristics of multiple sexual relationships and their association with youth risky sexual behaviour. All analysis was performed using the Statistical Package for Social Sciences (SPSS) version 22. The findings were presented by gender and residence in order to observe the variation among respondents.

## Study Variables

Selected variables were extracted from the Demographic Health Survey on sexual behaviour, total lifetime number of sexual partners, age group, marital status, religion, ethnicity, regions and place of residence. Number of sexual partners was redefined (one or less sexual partner versus more than one sexual partner). Other variables were defined as follows: age group was stratified into two age groups spanning five years (15–19 and 20–24 years), place of residence was defined as urban or rural, education level was defined as; no education, primary, secondary and higher, and regions (northern, central and southern). Wealth was stratified into poorest, poorer, middle, richer and richest, marital status was redefined as married, never married, and religion redefined as other Christian, catholic, Church of Central Africa Presbyterian (CCAP), Muslims, others. The category others included Anglican, Seventh day Adventist / Baptist and no religion. Ethnicity was redefined into Chewa, Lomwe, Ngoni, Tumbuka, Yao and others. The category others included; Tonga, Sena, Nkhonde, Other; Lambya, Other; Ndali, Other; Mang’anja, and Other; Nyanja.

## Statistical Analysis

The extracted data for males and females were weighted so that the sample was representative of 15–24 years respondents in the 2010 Demographic Health Survey. Analysis was performed using SPSS version 22, which accounted for the sample strata, the primary sampling unit and population weights. A descriptive statistic of youth’s number of sexual partners, stratified by gender and residence, was presented. Chi-square comparisons were conducted to identify differences between males and females' number of sexual partners and this was performed based on the socio-economic and demographic characteristics. A binary logistic regression model was then used without an offset to examine the relationship between number of sexual partners and the socio-economic and demographic characteristics. These models provided an estimate of the prevalence ratio for the relevant outcome. All analyses presented were stratified by gender and residence regardless of the significance of any interactions. However, one model was created by gender and residence. The model examined whether the difference in number of sexual partners was associated with young people’s risky sexual behaviours. Variables that were significant in the regression model were considered as a contributing factor to multiple sexual partnerships among youths which was considered as a high risk factor for STIs.

## Ethical Considerations

This study used secondary data from the Malawi Demographic and Health Survey. Prior to using these data, agreement was obtained from Macro International, which has allowed us to download the data on their Web Site. Please note that all data are fully available without restriction.

## Results

A total of 2026 males and 2290 females were considered in the study, and of these 1399 (69%) males and 2290 (35.4%) females reported multiple sexual partnerships (MSP) (Table 1). Among males, age group, religion, marital status and condom use were statistically significant. Having multiple sex partners was significant among males aged 20–24 years (75%, p<0.000), who were Muslims (80.3%, p<0.000), being married (74.3%, p<0.003), from the Yao (78.6%, p<0.000), and reported condom use (83.7%) during the last sexual debut. Among females, the prevalence of MSP was common among 20–24 years (37.7%, p<0.000), in the urban areas (42.3%, p<0.000), in the southern region (44.3%, p<0.000), from the richest households (38.6%, p<0.010), and being Muslims (47.9%, p<0.000). Never married females (38.2%, p<0.003), from the Yao (49.3%, p<0.000) ethnic group, who reported condom use (40.0%, p<0.007) reported a higher prevalence rate.

**Table 1:** Percentage of rural/urban males and females with multiple sexual partners by background characteristics.

Background characteristics					RURAL		URBAN	
	MALE		FEMALE		MALE	FEMALE	MALE	FEMALE
	Had multiple sex partner (n = 1399)	Weighted number of males (N = 2026)	Had multiple sex partner (n = 2290)	Weighted number of females (N = 6470)	Had multiple sex partner (n = 1082)	Had multiple sex partner (n = 1779)	Had multiple sex partner (n = 316)	Had multiple sex partner (n = 511)
***Age group***	***P = 0*.*000***		***P = 0*.*000***					
15–19	591 (62.3)	949	679 (30.9)	2197	464 (78.5)	524 (77.2)	127 (21.5)	155 (22.8)
20–24	807 (75.0)	1076	1611 (37.7)	4272	618 (76.6)	1255 (77.9)	189 (23.4)	356 (22.1)
***Residence***	***P = 0*.*828***		***P = 0*.*000***					
Urban	316 (69.5)	455	511 (42.3)	1208	-	-	-	-
Rural	1082 (68.9)	1570	1780 (33.8)	5262	-	-	-	-
***Region***	***P = 0*.*184***		***P = 0*.*000***					
Northern	122(67.0)	182	212 (27.6)	769	113 (93.4)	185 (87.3)	8 (6.6)	27 (12.7)
Central	604(67.3)	897	722 (27.4)	2639	470 (77.8)	543 (75.3)	134 (22.2)	178 (24.7)
Southern	673(71.1)	947	1356 (44.3)	3061	499 (74.1)	1051 (77.5)	174 (25.9)	305 (22.5)
***Education***	***P = 0*.*105***		***P = 0*.*088***					
No education	40 (69.0)	58	166 (37.5)	443	33 (82.5)	151 (91.0)	7 (17.5)	15 (9.0)
Primary	914 (70.8)	1291	1598 (35.6)	4487	784 (85.8)	1357 (84.9)	130 (14.2)	241 (15.1)
Secondary	410 (65.3)	628	484 (33.5)	1444	258 (63.1)	265 (54.6)	151 (36.9)	220 (45.4)
Higher	35 (71.4)	49	43 (44.3)	97	7 (20.0)	7 (16.7)	28 (80.0)	35 (83.3)
***Wealth***	***P = 0*.*181***		***P = 0*.*010***					
Poorest	225 (71.7)	314	417 (34.3)	1217	215 (95.6)	410 (98.6)	10 (4.4)	6 (1.4)
poorer	264 (67.9)	389	463 (34.2)	1354	258 (97.7)	448 (96.8)	6 (2.3)	15 (3.2)
Middle	279 (72.1)	387	440 (32.8)	1341	257 (92.4)	412 (93.6)	21 (7.6)	28 (6.4)
Richer	284 (70.0)	406	445 (37.1)	1198	217 (76.4)	324 (72.8)	67 (23.6)	121 (27.2)
Richest	347 (65.5)	530	525 (38.6)	1359	135 (38.9)	185 (35.2)	212 (61.1)	340 (64.8)
***Religion***	***P = 0*.*000***		***P = 0*.*000***					
Other christian	448 (67.7)	662	913 (35.7)	2558	363 (81.0)	706 (77.3)	85(19.0)	207(22.7)
Catholic	258 (61.4)	420	404 (31.2)	1294	208 (80.3)	311 (77.0)	51 (19.7)	93(23.0)
CCAP	262 (67.4)	389	300 (29.0)	1034	190 (72.8)	217 (72.3)	71(27.2)	83(27.7)
Muslim	241 (80.3)	300	462 (47.9)	964	180 (74.7)	389 (84.0)	61(25.3)	74(16.0)
Others	189 (74.4)	254	211 (34.1)	619	141 (74.6)	157 (74.4)	48(25.4)	54(25.6)
***Marital status***	***P = 0*.*003***		***P = 0*.*003***					
Married	375 (74.3)	505	1588 (34.3)	4634	313 (83.5)	1273 (80.2)	62(16.5)	315(19.8)
Never married	1023 (67.3)	1520	702 (38.2)	1836	769 (75.2)	506 (72.1)	254(24.8)	196(27.9)
***Ethnicity***	***P = 0*.*000***		***P = 0*.*000***					
Chewa	420 (64.1)	655	583 (27.6)	2113	357 (85.0)	469 (80.4)	63(15.0)	114(19.6)
Lomwe	253 (70.7)	358	451 (42.2)	1069	197 (77.9)	348 (77.2)	56(22.1)	103(22.8)
Tumbuka	99 (61.9)	160	310 (38.8)	800	85 (85.9)	207 (66.8)	14(14.1)	103(33.2)
Ngoni	196 (71.0)	276	153 (25.8)	592	130 (66.3)	121 (79.1)	66(33.7)	32(20.9)
Yao	232 (78.6)	295	483 (49.3)	979	166 (71.6)	385 (79.7)	66(28.4)	98(20.3)
Others	199 (70.6)	282	311 (33.9)	918	147 (74.2)	250 (80.4)	51(25.8)	61(19.6)
***Condom use***	***P = 0*.*000***		***P = 0*.*007***					
No	1276 (67.9)	1879	2007 (34.8)	5760	771 (78.8)	1609 (80.2)	119(22.7)	398(19.8)
Yes	123 (83.7)	147	284 (40.0)	710	311 (74.0)	171 (60.2)	20(19.4)	113(39.8)

Source: Computed by the authors

Within the rural area, females (n = 1779) were more likely to report MSP than males (n = 1082). Most males who reported MSP were aged 15–19 years (78.5%), in the northern region (93.4%), with primary education (85.8%), in the poorer household (97.7%), other Christian (81.0%), who are married (83.5%), from the Tumbuka (85.9%) ethnic group, and did not use condoms (78.8%) during the last sexual encounter. About 78% rural females with multiple sex partners were aged 20–24 years, (91%) had no education, about 99% from the poorest households, being Muslim (84%), who were married (80.2%), from the Chewa (80.4%) ethnic group, and did not use condoms (80.2%) during the last sexual encounter.

Within the urban area, a higher proportion of females (n = 511) still reported MSP than males (n = 316). Most males who reported MSP were aged 20–24 years (23.4%), in the southern region (25.9%), with higher education (80%) and from the richest (61.1%) households. Moreover, CCAP males (27.2%), who were never married (24.8%), from the Ngoni (33.7%) ethnic group, who did not use condoms (22.7%), also reported MSP. The study therefore found that, most respondents who reported MSP within the rural area were in the northern region, who were married, and did not use condoms. Meanwhile, within the urban area, respondents who reported MSP had a higher education, from the richest households, being CCAP members who were never married.

Table 2 presents the adjusted odds ratios and rural / urban variation of multiple sexual partnerships among youths in Malawi. The prevalence of MSP was high among females from Lomwe (OR = 1.32), Ngoni (OR = 1.57) and Yao (OR = 1.62), with no education (OR = 1.21). Meanwhile, the odds was lower among females aged 15–19 years (OR = 0.64), who were married (OR = 0.79), and did not use condoms (OR = 0.82). Among males in the poorest (OR = 1.49) and middle household (OR = 1.66) income, who did not use condoms (OR = 2.63), the prevalence of MSP was higher. Those aged 15–19 years (OR = 0.48), that were Catholic (OR = 0.51) and from the Chewa (OR = 0.66) ethnic group were less interested in MSP. Rural males with primary education (OR = 4.49), in the middle household income (OR = 1.66), who did not use condoms (OR = 2.63) were more likely to have multiple sex partners. While those aged 15–19 years (OR = 0.48), that were Catholic (OR = 0.43) were less likely to have MSP.

**Table 2:** Adjusted odds ratios of rural/urban males and females aged 15–24 years with multiple sexual partners by background characteristics.

Background characteristics	FEMALE	MALE	RURAL		URBAN	
			MALE	FEMALE	MALE	FEMALE
	Odds ratios (95% C.I)	Odds ratios (95% C.I)	Odds ratios (95% C. I)	Odds ratios (95% C.I)	Odds ratios (95% C.I)	Odds ratios (95% C. I)
***Age group***						
15–19	0.64***	0.48***	0.48***	0.61***	0.39***	0.64***
20-24(ref)	**1.00**	**1.00**	**1.00**	**1.00**	**1.00**	**1.00**
***Residence***						
Urban	1.39***	1.19	-	-	-	-
Rural (ref)	**1.00**	**1.00**	-	-	-	-
***Religion***		***	***	*		*
Other christian	1.15	0.66*	0.57**	1.06	1.07	0.82
Catholic	1.01	0.51***	0.43***	0.94	1.09	0.69
CCAP	0.98	0.75	0.65	0.92	0.70	0.80
Muslim	1.29	1.33	1.38	1.52	1.35	1.18
Others (ref)	**1.00**	**1.00**	**1.00**	**1.00**	**1.00**	**1.00**
***Region***	***			***		***
Northern	0.72**	0.97	0.79	0.77	1.87	1.01
Central	0.52***	1.07	0.86	0.54***	1.89*	1.74***
Southern (ref)	**1.00**	**1.00**	**1.00**	**1.00**	**1.00**	**1.00**
***Education***	**	**	**	**		*
No education	1.21*	0.86	3.01	1.49	0.81	0.39*
Primary	1.22	1.23	4.49*	1.59	0.54	0.68
Secondary	0.93	0.79	2.98	1.23	0.33*	1.01
Higher (ref)	**1.00**	**1.00**	**1.00**	**1.00**	**1.00**	**1.00**
***Wealth***		*			*	***
Poorest	0.94	1.49*	1.30	1.17	3.57	3.45***
Poorer	0.95	1.17	1.06	1.09	0.40	2.73**
Middle	0.88	1.66**	1.57*	1.06	0.71	1.87**
Richer	1.03	1.25	0.98	0.97	2.93**	1.17
Richest (ref)	**1.00**	**1.00**	**1.00**	**1.00**	**1.00**	**1.00**
***Marital status***						
Married	0.79***	1.07	0.59	0.77***	3.12**	4.22***
Never married (ref)	**1.00**	**1.00**	**1.00**	**1.00**	**1.00**	**1.00**
***Ethnicity***	***	*	*	***		
Chewa	1.15	0.66*	0.79	1.07	0.39	0.58**
Lomwe	1.32**	1.22	1.30	1.36**	0.55	0.82
Ngoni	1.57***	0.67	0.92	1.49**	0.29*	0.66*
Tumbuka	0.92	1.04	1.59	0.83	0.31**	0.72
Yao	1.62***	0.98	1.06	1.39*	0.63	0.57*
Others (ref)	**1.00**	**1.00**	**1.00**	**1.00**	**1.00**	**1.00**
***Condom use***						
No	0.82*	2.63***	2.27***	0.89	4.88***	0.64*
Yes (ref)	**1.00**	**1.00**	**1.00**	**1.00**	**1.00**	**1.00**

Source: Malawi Demographic and Health Survey 2010,

***p<0.001,

**p<0.01,

*p<0.05,

(ref) = reference category

Among rural females, those from the Lomwe (OR = 1.36) and Ngoni (OR = 1.49) ethnic groups were more likely to have MSPs, while those aged 15–19 years (OR = 0.61), in the central region (OR = 0.54), who were married (OR = 0.77) were less likely to have MSPs. Urban males in the central region (OR = 1.89) from the richer households (OR = 2.93) that were married (OR = 3.12) and did not use condoms (OR = 4.88) were more likely to report MSP. Meanwhile, those that were aged 15–19 years (OR = 0.39) with secondary education (OR = 0.33) and from the Ngoni (OR = 0.29) and Tumbuka (OR = 0.31) ethnic groups reported a lower prevalence rate of MSPs.

Urban females in the central region (OR = 1.74) from the poorest households (OR = 3.45) and being married (OR = 4.22) were more likely to have MSP. While those that were aged 15–19 years (OR = 0.64) with no education (OR = 0.39) from the Chewa (OR = 0.58) ethnic group and did not use condoms (OR = 0.64) were less likely to report MSPs.

## Discussion

This study examines the associated risk factor of STIs and multiple sexual relationships among youths in Malawi. The study has shown that, the prevalence of multiple sexual partnerships was more common among females than males. The prevalence among males is lower than what other studies have reported. In a follow up study in Zimbabwe, multiple sexual partnerships (MSP) prevalence of 40% for males and 6% females was reported [39] and a recent study of community norms and attitudes towards multiple and concurrent sexual partnership reported a prevalence of 37.1% for males and 7.3% for females [40]. Researchers have therefore emphasized that, more attention be paid to the role of concurrent sexual partnerships when studying issues regarding the spread of HIV/STIs [41]. Others found that youths aged 15–19 years old were more likely to report multiple sexual partnerships than those aged 20–24 years [42] thus increasing the risk for HIV/STIs.

Whether wealth is a protective or risk factor for multiple sexual relationships was given some consideration in the study. Based on the current study, the prevalence of multiple sexual partnerships was higher among males in the poorest and middle wealth quintiles. Meanwhile, urban females in the richer quintile were more likely to engage in multiple sexual relationships. Researchers have reported mixed findings with a higher risk of HIV and other STIs among women in the middle and second richest wealth quintiles in Malawi [43]. Others have described the attitudes of young males and females toward sexuality as an ‘effort to survive’ in the face of poverty [44]. Others have described it as a struggle against poverty, a quest for survival and consumerism as the driver for multiple sexual partnerships in Malawi [45]. Having more than one sexual partner was found to be a risk factor for STIs among youths in Malawi. Females in the urban areas were more likely to have more sexual partners than those in the rural areas and more than males from both rural and urban areas.

Furthermore, marriage was also given some attention in the study. Among men who have sex with other men in China, it was reported that, 80% of those who never tested for STIs, were never married compared with 62.2% who tested [46]. In Malawi, 86% of sexually active youths reported 0 or 1 partner, while 5% reported multiple consecutive partnerships [47]. The results from the current study indicate that MSP prevalence was higher among married males and lower among females and 4.2 times higher among urban females. The observed differences by gender and residence could possibly be a result of differences in the socio-cultural practices across regions in the study area.

Studies have shown that, youths engage in multiple sexual partnerships because of dissatisfaction with their partner’s sexuality or otherwise in addition to lack of communication and romance among partners, lack of skills in love making and desire for variety in partners and sex [48]. Moreover, youths have done so in order to have more opportunities in choosing who they will marry as they grow up and by so doing exposing themselves to the risk of Sexually Transmitted Infections. Being married predisposes one to take preventive measures against the spread of Sexually Transmitted Infections, because never married youths are repeatedly exposed to unprotected sex, unwanted pregnancy, and unsafe abortion at a youthful age which increases the risk of contracting STIs [49]. Furthermore, dissatisfaction with stable relationships, financial dissatisfaction, emotional and sexual dissatisfaction was a contributing factor toward multiple sexual relationships in Tanzania [50]. Education was also given some consideration in the study. According to the study findings, rural males with primary education, and females with no education were more likely to have MSP. Moreover, urban females with no education were less likely to engage in multiple sexual relationships. Studies have shown that youths stay protected from the risk of HIV infection and other STIs when they stay in school [51]. More years of schooling are associated with a relatively late initiation of sex, especially among females and it thus reduces premarital and recent sexual intercourse for females. Among males, evidence of STIs risk was found if one belongs to certain religious groups. Male who were other Christian and Catholic had greater odds of having multiple sex partners. Moreover, the prevalence of multiple sexual relationships was higher among Catholic and other Christian males. Certain religious beliefs allow individuals to have more than one sexual partner, thus facilitating the spread of Sexually Transmitted Infections. Within the Islamic communities of Machinga and the neighbouring districts, having more than one sexual partner was accepted by religion but the consequences of multiple sexual partners as a risk factor for sexually transmitted infections was not studied [52]. In another study of rural women in Tanzania, being a Muslim woman increases the odds of MSP by 1.56 times than that for Christian women [53]. Moreover, among Christian youths in the Wakiso district of Uganda, those with less interest in religion were more likely to have higher HIV infections since they are more likely to engage in MSP [54]. Evidence of STIs risk was found by age group, especially among females. Irrespective of the fact that having one sexual partner reduces the risk of sexually transmitted infections, a greater proportion of youths in the study area remain at risk of contracting sexually transmitted infections and unintended pregnancies. This is evident from the present study which indicates that among sexually active youths, 58.4% females aged 20–24 years had sex with more than one partner, while about 22% males of the same age group had sex with more than one partners. This study has shown that having more than one sexual partner among youths is an associated risk factor of sexually transmitted infections among youths, with females at greater risk than males. Multiple life time partners therefore lead to greater risk of STIs than the same number of sequential multiple partners. Having multiple life time partners in a dense sexual network increases the risk of sexually transmitted infections, thus allowing the virus to spread rapidly. It is therefore recommended that, more attention be placed on the role of having more than one sexual partner when studying issues pertaining to the risk of sexually transmitted infections.

## Conclusion

Multiple sexual relationships are associated with STIs and other sexual and reproductive health problems such as unwanted pregnancy and unsafe abortion among youths in Malawi. The study suggests the need for further research and prevention programs in order to educate youths on sexual and reproductive health issues which remain one of the most important strategies to address risky sexual behaviours such as multiple sexual relationships among youths. Although the prioritization of reproductive health services and youth’s education on the consequences of multiple sexual relationships might halt the spread of STIs, abstinence has always remained the most reliable method of prevention against the risk of STIs.

## Supporting Information

S1 File Demographic and Health Surveys data agreement. (DOCX)
